# Vascularized fibular graft versus non-vascularized graft after tumor resection

**DOI:** 10.6026/973206300220456

**Published:** 2026-01-31

**Authors:** Shivvir Singh, Atul Solankil, Anil Vaishnav

**Affiliations:** 1 Department of Orthopaedics, Virendra Kumar Sakhlecha Government Medical College, Neemuch, Madhya Pradesh, India; 2 Department of Orthopaedics, Government Medical College, Datia, Madhya Pradesh, India

**Keywords:** Vascularized fibular graft, non-vascularized graft, tumor resection, bone reconstruction, MSTS score

## Abstract

Segmental bone defects from tumor resection require effective reconstructive strategies. Therefore, it is of interest to compare vascularized fibular grafting (VFG) and non-vascularized fibular grafting (NVFG) in 60 patients. VFG showed better union rates, earlier consolidation and superior graft remodeling. Functional outcomes, measured by MSTS scores, favored VFG. Although complications were similar, non-union and revision procedures were more frequent in the NVFG group. Thus, we show the advantages of VFG for reliable bone defect reconstruction.

## Background:

Reconstruction of segmental long-bone defects after oncologic resection remains a fundamental challenge in limb-salvage orthopaedic oncology. Biological reconstruction aims to restore durable bone stock and maintain long-term function in predominantly young patients and fibular auto grafts - both vascularized and non-vascularized - are widely used options for this purpose. Bibliometric and clinical literature indicate sustained global interest in fibular grafting and an expanding array of techniques (single/ double-barrel, composite constructs with allograft or devitalized auto graft and virtual planning) tailored to defect size and location. Recent trends emphasize vascularized fibular techniques for large or load-bearing reconstructions because of their biological advantages [1]. Vascularized fibular grafting (VFG) provides a living bone transfer that preserves periosteal and end osteal blood supply, which theoretically accelerates incorporation, reduces time to union and permits progressive hypertrophy under mechanical loading. Several series and comparative studies support faster and more reliable union with VFG, particularly for long defects and when used in isolation or in composite constructs. In a comparative series of fibular reconstructions after tumor resection, vascularized free fibular flaps were associated with higher union rates than non-vascularized grafts, although functional scores were not uniformly different between techniques [2]. Hypertrophic adaptation of vascularized fibulae - an important mechanism by which the graft attains mechanical competence has been quantified, with early periosteal hypertrophy followed by endosteal remodeling over months to years [3]. Large single-centre and multi-institutional cohorts have documented generally favorable union rates and acceptable complication profiles after VFG and contemporary reports describe satisfactory functional outcomes (MSTS scores) across varied indications. Nevertheless, results are heterogeneous: some investigators report no significant difference in union time or function between vascularized and non-vascularized fibular grafting when grafts are used as part of composite reconstructions (*e.g.*, fibula inserted into an allograft or devitalized autograft), suggesting that the choice of technique may interact with fixation strategy, defect location and adjunctive constructs [4, 5- 6]. Therefore, it is of interest to evaluate radiological union, time to consolidation, graft remodeling, functional recovery and complication rates following vascularized versus non-vascularized fibular reconstruction after segmental tumor resection.

## Materials and Methods:

## Study design and setting:

This investigation was structured as a prospective, comparative cohort study conducted in a tertiary referral center. Written informed consent was secured from all participants or guardians where applicable.

## Study population:

Patients undergoing segmental bone resection for primary benign-aggressive or low- to intermediate-grade malignant bone tumors of the femur, tibia, humerus or radius were screened for eligibility.

## Inclusion criteria:

[1] Age 12-55 years

[2] Requirement of segmental resection with a bone defect ≥6 cm

[3] Tumors with clear indication for graft reconstruction

[4] Ability to comply with follow-up for at least 24 months

## Exclusion criteria:

[1] High-grade malignancies requiring endoprosthetic reconstruction

[2] Pathological fractures with significant contamination

[3] Previous surgery or radiation to the affected limb

[4] Systemic vascular disorders precluding microvascular surgery

[5] Evidence of metastatic disease at presentation

## Sample size calculation:

Sample size was estimated to detect a minimum clinically relevant difference of 20% in union rates between groups, assuming a union rate of 80% in the vascularized graft group and 60% in the non-vascularized group, with α = 0.05 and power = 80%. This yielded a minimum requirement of 27 participants per arm. To accommodate potential loss to follow-up, the target enrollment was set at 60 patients (30 in each group).

## Grouping and allocation:

Participants were allocated to one of two groups based on the reconstructive technique selected by the senior surgical team:

[1] Group A: Vascularized fibular graft (VFG) - microvascularized free fibular graft used to reconstruct the defect.

[2] Group B: Non-vascularized fibular graft (NVFG) - conventional autologous fibular strut graft without micro vascular anastomosis. Allocation was not randomized due to anatomical and vascular suitability criteria; however, baseline characteristics were matched as closely as possible.

## Surgical technique:

## Group A - Vascularized fibular graft:

A free fibular graft with its nutrient artery was harvested, followed by microvascular anastomosis to local recipient vessels. The graft was contoured to bridge the resected segment and secured using either a locking plate or intramedullary fixation based on location.

## Group B - Non-vascularized fibular graft:

A standard fibular strut graft was harvested and inserted across the defect. Fixation was achieved using a plate-screw construct. No vascular anastomosis was performed.

In both groups:

[1] Tumor resection was executed with oncologically safe margins.

[2] Structural stability was ensured using similar fixation principles.

[3] Postoperative protocols were standardized with staged weight-bearing.

## Postoperative care and follow-up:

All participants underwent serial follow-up at 6 weeks, 3 months, 6 months, 12 months, 18 months and 24 months, with subsequent annual review. Each follow-up included:

[1] Clinical examination

[2] Radiographs of the reconstructed segment

[3] Functional assessment using the Musculoskeletal Tumor Society (MSTS) score

[4] Advanced imaging (CT/MRI) was performed when union was uncertain or if complications were suspected.

## Outcome measures:

## Primary outcome:

Radiological union time, defined as cortical continuity in at least three of four cortices.

## Secondary outcomes:

[1] Graft hypertrophy (for VFG) assessed via serial imaging

[2] Incidence of complications including graft fracture, infection, nonunion and hardware failure

[3] Revision surgery rate

[4] Functional outcome via MSTS score

## Statistical analysis:

Data analysis was executed using SPSS version 27. Categorical variables were expressed as frequencies and compared using the chi-square or Fisher's exact test. Continuous variables were examined using Student's t-test depending on distribution. Kaplan-Meier curves were used to evaluate union probability over time. A p-value <0.05 was considered statistically significant.

## Results:

The two groups demonstrated comparable baseline demographic and clinical characteristics, with no significant differences in age distribution, sex composition, tumor site patterns and tumor biology or resection length (Table 1). This similarity indicates that both cohorts were balanced at the time of enrollment, allowing for a reliable comparison of postoperative outcomes. A clear distinction in radiological performance was observed between the two reconstructive techniques. The vascularized graft cohort showed a higher likelihood of achieving union, along with a substantially shorter consolidation period. Evidence of graft remodeling at one year was also more prominent in this group and postoperative function, assessed using MSTS scoring, favored the vascularized reconstruction approach (Table 2). Evaluation of postoperative complications revealed fewer adverse events in the vascularized graft group. The incidence of non-union was notably lower, whereas other complications such as graft breakage, deep infection or hardware-related issues did not differ meaningfully between the groups. Although the requirement for revision procedures trended higher in the non-vascularized cohort, this difference did not reach statistical significance. Donor-site morbidity remained comparable in both groups (Table 3).

## Discussion:

In this prospective comparative cohort, both groups were well matched at baseline, which supports the internal validity of the outcome comparisons (Table 1). The equivalence in age, sex distribution, tumor location, tumor type and mean resection length indicates that differences observed in healing, remodeling and function are unlikely to be explained by measurable preoperative imbalance (Table 1). Our principal finding is that VFG produced superior biological performance: Higher rate of radiological union, shorter time to union and more frequent graft hypertrophy - accompanied by better limb function - when compared with NVFG (Table 2). The difference in union frequency and in consolidation time between groups was statistically and clinically important and mirrors outcomes reported in recent series and systematic syntheses that show high primary-union rates and relatively rapid consolidation after free vascularized fibular transfer in oncologic and large-defect reconstructions [7, 8]. The greater incidence of radiographic hypertrophy in the VFG cohort is consistent with the concept that a living, revascularized cortical graft can hypertrophy under load and progressively assume mechanical function, a mechanism repeatedly observed in contemporary clinical studies [9]. Complication profiles in our study favored the vascularized group with respect to non-union (Table 3), although several other adverse events (graft fracture, deep infection, hardware failure) did not differ significantly between groups. These findings are concordant with the heterogeneous literature: some comparative reports and reviews suggest that VFGs may reduce nonunion and improve functional indices in selected reconstructions, whereas other analyses have not found a consistent difference in overall complication or revision rates and have cautioned that vascularized procedures can carry a higher reoperation burden in specific series [10, 11]. Taken together, this suggests that while VFGs provide a biological advantage for incorporation and remodeling (reflected in our union, time-to-union and hypertrophy results), the net effect on complication or revision rates may depend on case-mix, graft length, fixation strategy and institutional experience [8, 10]. Donor-site morbidity in our cohorts was low and similar between groups (Table 3). Recent reports raise awareness that donor-site sensory changes, weakness or instability can occur after fibular harvest and that the incidence varies with the assessment method and length of follow-up; nevertheless, many series conclude that donor morbidity is acceptable for the functional gains of limb reconstruction when indicated [12, 13]. Thus, in appropriately selected patients the biological and functional benefits of VFG can be achieved without a prohibitive donor-site penalty.

There are several practical implications of these results. First, when reliable and timely union is a primary objective - for example, in young patients, weight-bearing lower-limb reconstructions or isolated fibular reconstructions without supportive composite constructs - a vascularized fibula should be strongly considered because it shortens time to structural consolidation and promotes hypertrophic remodeling (Table 2). Second, surgeons should weigh the technical demands, operative time and resources required for microvascular transfer against the biological advantages; institutional experience influences both graft survival and complication rates and likely contributed to the favorable outcomes observed here. Lastly, fixation technique and adjuncts (composite allograft, double-barrel constructs or additional cancellous grafting) interact with graft biology and may modify the comparative performance of VFG and NVFG; therefore, reconstructions should be planned as integrated construct-biology solutions rather than as decisions about graft type alone [7, 8]. Study limitations deserve emphasis. This was a non-randomized cohort and although baseline variables were comparable (Table 1), unmeasured confounding is possible. The sample size, while adequate to detect the observed differences in union and time to consolidation, limits precise estimates for less frequent complications and for subgroup analyses by location or graft length. Finally, longer follow-up would strengthen conclusions about late complications, hypertrophy maturation and long-term function. Future multicenter, preferably randomized, studies or large registry analyses that report individual graft lengths, fixation details and standardized functional outcomes would be valuable to refine selection criteria for VFG versus NVFG [10, 11].

## Conclusion:

Vascularized fibular grafting demonstrated superior biological and functional performance compared with non-vascularized fibular grafts following segmental bone reconstruction after tumor resection. Although both groups were similar at baseline, the vascularized technique was associated with higher union rates, faster consolidation and greater graft remodeling and better functional recovery. Complication patterns also favored the vascularized approach, particularly with respect to non-union, without increasing donor-site morbidity. These findings support the preferential use of vascularized fibular grafts in cases requiring reconstruction of large post- resection defects, especially when rapid and reliable integration is desired. Further studies with longer follow-up may help clarify long-term durability and the impact on limb function and quality of life.

## Figures and Tables

**Table 1:** Baseline demographic and clinical characteristics

**Variable**	**Vascularized Fibular Graft (VFG) (n = 30)**	**Non-Vascularized Fibular Graft (NVFG) (n = 30)**	**p-value**
Mean age (years)±SD	28.6±10.4	29.8±11.1	0.68
Sex (M/F)	17 / 13	18 / 12	0.79
**Tumor location**			
- Femur	11 (36.7%)	12 (40%)	0.91
- Tibia	10 (33.3%)	9 (30%)	
- Humerus	6 (20%)	5 (16.7%)	
- Radius	3 (10%)	4 (13.3%)	
**Tumor type**			
- Benign-aggressive	13 (43.3%)	14 (46.7%)	0.87
- Low-grade malignancy	10 (33.3%)	9 (30%)	
- Intermediate-grade malignancy	7 (23.3%)	7 (23.3%)	
Mean resection length (cm)±SD	8.7±1.9	8.4±2.1	0.56

**Table 2:** Radiological and functional outcomes

**Outcome**	**VFG (n = 30)**	**NVFG (n = 30)**	**p-value**
Radiological union achieved	28 (93.3%)	22 (73.3%)	0.04
Mean time to union (months) ± SD	7.4±1.8	10.6±2.3	<0.001
Graft hypertrophy at 12 months	21 (70%)	4 (13.3%)	<0.001
MSTS functional score (mean±SD)	25.8±2.1	22.4±3.0	<0.001

**Table 3:** Complications and revisions

**Complication / Event**	**VFG (n = 30)**	**NVFG (n = 30)**	**p-value**
Non-union	2 (6.7%)	8 (26.7%)	0.03
Graft fracture	1 (3.3%)	4 (13.3%)	0.16
Deep infection	1 (3.3%)	2 (6.7%)	0.55
Hardware failure	1 (3.3%)	3 (10%)	0.3
Revision surgery required	2 (6.7%)	7 (23.3%)	0.07
Donor-site morbidity	3 (10%)	2 (6.7%)	0.64
